# Editorial: Cutaneous lupus erythematosus landscape: pathophysiology, unmet needs, and related challenges in clinical practice. What is on the horizon?

**DOI:** 10.3389/fmed.2024.1373552

**Published:** 2024-02-27

**Authors:** Joseph F. Merola, Filippa Nyberg, Nathalie Franchimont, Catherine Barbey, Victoria P. Werth

**Affiliations:** ^1^Department of Dermatology and Department of Medicine, UT Southwestern Medical Center, Dallas, TX, United States; ^2^Department of Dermatology, Karolinska University Hospital, Stockholm, Sweden; ^3^Biogen, Cambridge, MA, United States; ^4^Biogen, Baar, Switzerland; ^5^Department of Dermatology, University of Pennsylvania and Philadelphia VAMC, Philadelphia, PA, United States

**Keywords:** cutaneous lupus erythematosus (CLE), plasmacytoid dendritic cell (PDC), skin manifestation, autoimmune diseases, quality of life

Cutaneous lupus erythematosus (CLE) is an autoimmune disease with heterogeneous skin manifestations that can occur with or without systemic manifestations with no approved drug(s) specifically for the treatment of CLE. Authors of this Research Topic provide an overview of the current landscape and the emerging understanding of CLE as a distinct autoimmune entity. They highlight future directions and obstacles that should be addressed to advance targeted therapies in CLE.

Investigation of the incidence and prevalence of CLE in the absence of systemic lupus erythematosus (SLE) has been limited to date (Walker et al.). Current epidemiological studies suggest that race and ethnicity do affect CLE diagnosis frequency: discoid lupus erythematosus (DLE) occurs more frequently in Black or Hispanic patients and subacute cutaneous lupus erythematosus (SCLE) occurs more frequently in White populations. Both the severity and disease course can differ by race for CLE. For instance, Black patients have been shown to have higher baseline disease damage [as measured by the Cutaneous Lupus Erythematosus Disease Area and Severity Index (CLASI) instrument] than non-Black patients, and strong correlation was found between CLASI-damage and activity score in Black while there were no correlation in White patients. Disease activity has been shown to impact Quality of Life (QoL) (1). Additionally, socio-demographic factors contribute to overall outcomes in CLE patients with income, educational background, and access to health insurance among the contributing factors. Additional epidemiological data and analyses of CLE disease burden across race and ethnicity are needed and diversity inclusiveness is warranted in CLE studies (Walker et al.).

The pathophysiology of CLE resembles and overlaps with that of SLE. Many factors have been proposed to trigger immune responses in CLE including genetic predisposition, environmental factors such as ultraviolet irradiation, and certain pharmaceutical agents (Chen et al., Klein and Kunz, Fetter et al.). Multiple genes responsible for mediating innate immune response, cell growth, apoptosis, and interferon response as well as increased frequency of HLA-B8 and C2 complement deficiency have been identified as genetic aberrations or transcript anomalies linked to CLE (Chen et al.). Numerous mechanisms driven by ultraviolet light have been identified to contribute to the pathogenesis of CLE (Klein and Kunz). These mechanisms result in the chronic activation of immune pathways, which is considered a hallmark mechanism of CLE pathophysiology and is characterized by the production of type I interferon (IFN-I) (Fetter et al.).

CLE has three major subtypes: SCLE, acute (ACLE), and chronic (CCLE), and patients may exhibit more than one subtype at a time (Elmgren and Nyberg). Elmgren and Nyberg reviewed the association of CLE with SLE noting that while many shared features point to CLE and SLE as being part of a disease spectrum, current evidence suggests that they are closely related but distinct diseases with different courses. Histopathologically, CLE is characterized by the presence of lymphocytic infiltrates and necroptotic keratinocytes at the dermo-epidermal junction. However, CLE subtypes are heterogeneous in their clinical appearance, and histopathological features. Fetter et al. summarize the histopathological features characteristic of the different CLE subtypes in their review, acknowledging that the overlap in histology often does not allow a clinical subset diagnosis from histology and highlights the importance of knowing the specific subtype molecular signature to develop a precision medicine approach to CLE treatment.

There are no drugs approved specifically for the treatment of CLE to date. Current treatment guidelines for CLE recommend a combination of preventive measures and topical and systemic medications (Verdelli et al.). First-line treatment may include topical corticosteroids and systemic antimalarials. Second- and third-line systemic treatments include immunosuppressants and immunomodulatory drugs. Targeted biologics approved for the treatment of SLE may be available to the subset of patients with CLE. Despite recommended treatment guidelines, approximately 10% of CLE patients have been shown to be refractory to therapy (2). As such, there is a clear need to develop targeted therapies specifically for CLE.

Development of novel CLE specific therapies is currently a growing area, but is complicated by the lack of standardized outcome measures to be used in clinical trials. Gaffney et al. summarize a working core domain set and core outcome set for CLE recommended for use in clinical trials as an interim guide until standardized outcomes are fully available (3). The authors discussed currently available and new clinical outcomes, such as the CLASI scale and/or a CLE-specific investigator global assessment of disease activity (CLA-IGA). They also reviewed patient reported outcomes, and QoL measures such as the Skindex-29+3 and the CLE-QoL. Not all of these outcome measures have been validated in CLE highlighting future requirements for additional work in this area (Gaffney et al.).

Although no therapies have been approved specifically for the treatment of CLE, several emerging therapies are under investigation (Sprow et al.). Although anifrolumab and belimumab were previously not studied in CLE specific trials, analyses of skin focused outcome measures of SLE patients with skin manifestations showed improvement in the treatment groups over placebo suggesting promise of these agents for CLE. CLE specific studies are currently underway with agents targeting various pathways such as the IFN-alpha receptor (anifrolumab), plasmacytoid dendritic cell [litifilimab and daxdilimab], TYK-2 (deucravacitinib), toll-like receptor [enpatoran, and interleukin-1 receptor-associated kinase 4 (IRAK4) (edecesertib)]. Litifilimab and daxdilimab have previously demonstrated clinical benefit in some forms of CLE but need to be investigated in larger and longer trials (4, 5).

Patients living with CLE experience poor QoL, particularly in the psychological and social health domains (Drenkard et al.). Many factors have been reported to negatively impact health-related QoL (HRQoL) for CLE patients including female sex, low education, and higher skin disease activity among others. Pain, fatigue, disease activity, body image, and medication side effects are specific areas that CLE patients have reported impacting their QoL. With regards to the psychological domain, CLE patients have an increased prevalence of major depressive disorder, generalized anxiety disorder, panic disorder, suicide risk, and agoraphobia. Altogether, these observations highlight the need to provide new therapeutic solutions for CLE patients that would improve their QoL.

The current landscape of CLE presents many areas of opportunity for the scientific and healthcare community to pursue including improving diagnoses, identifying and understanding the molecular mechanisms driving disease, developing novel therapies to target these mechanisms. These endeavors should be considered in a patient-centric approach to ultimately improve patient outcomes and QoL for those living with CLE.

## Author contributions

JM: Writing—original draft, Writing—review & editing. FN: Writing—original draft, Writing—review & editing. NF: Writing—original draft, Writing—review & editing. CB: Writing—original draft, Writing—review & editing. VW: Writing—original draft, Writing—review & editing.

## References

[1] Verma SM Okawa J Propert KJ Werth VP. The impact of skin damage due to cutaneous lupus on quality of life. Br J Dermatol. (2014) 170:315–21. 10.1111/bjd.12653 24111880 PMC4878997

[2] Moghadam-Kia S Chilek K Gaines E Costner M Rose ME Okawa J. Cross-sectional analysis of a collaborative web-based database for lupus erythematosus–associated skin lesions: prospective enrollment of 114 patients. Arch Dermatol. (2009) 145:255–60. 10.1001/archdermatol.2008.594 19289753 PMC2830744

[3] Guo LN Perez-Chada LM Borucki R Nambudiri VE Werth VP Merola JF. Development of a working core outcome set for cutaneous lupus erythematosus: a practical approach to an urgent unmet need. Lupus Sci Med. (2021) 8:e000529. 10.1136/lupus-2021-000529 34969875 PMC8718411

[4] Werth VP Furie RA Romero-Diaz J Navarra S Kalunian K van Vollenhoven RF. Trial of anti-BDCA2 antibody litifilimab for cutaneous lupus erythematosus. N Engl J Med. (2022) 387:321–31. 10.1056/NEJMoa2118024 35939578

[5] Karnell JL Wu Y Mittereder N Smith MA Gunsior M Yan L. Depleting plasmacytoid dendritic cells reduces local type I interferon responses and disease activity in patients with cutaneous lupus. Sci Transl Med. (2021) 13:eabf8442. 10.1126/scitranslmed.abf8442 34039741

